# Role of serotonergic neurons in the *Drosophila *larval response to light

**DOI:** 10.1186/1471-2202-10-66

**Published:** 2009-06-23

**Authors:** Verónica G Rodriguez Moncalvo, Ana Regina Campos

**Affiliations:** 1 Department of Biology, McMaster University, 1280 Main St. West, Hamilton, ON, L8S 4K1, Canada

## Abstract

**Background:**

*Drosophila *larval locomotion consists of forward peristalsis interrupted by episodes of pausing, turning and exploratory behavior (head swinging). This behavior can be regulated by visual input as seen by light-induced increase in pausing, head swinging and direction change as well as reduction of linear speed that characterizes the larval photophobic response. During 3^rd ^instar stage, *Drosophila *larvae gradually cease to be repelled by light and are photoneutral by the time they wander in search for a place to undergo metamorphosis. Thus, *Drosophila *larval photobehavior can be used to study control of locomotion.

**Results:**

We used targeted neuronal silencing to assess the role of candidate neurons in the regulation of larval photobehavior. Inactivation of DOPA decarboxylase (Ddc) neurons increases the response to light throughout larval development, including during the later stages of the 3^rd ^instar characterized by photoneutral response. Increased response to light is characterized by increase in light-induced direction change and associated pause, and reduction of linear movement. Amongst Ddc neurons, suppression of the activity of corazonergic and serotonergic but not dopaminergic neurons increases the photophobic response observed during 3^rd ^instar stage. Silencing of serotonergic neurons does not disrupt larval locomotion or the response to mechanical stimuli. Reduced serotonin (5-hydroxytryptamine, 5-HT) signaling within serotonergic neurons recapitulates the results obtained with targeted neuronal silencing. Ablation of serotonergic cells in the ventral nerve cord (VNC) does not affect the larval response to light. Similarly, disruption of serotonergic projections that contact the photoreceptor termini in the brain hemispheres does not impact the larval response to light. Finally, pan-neural over-expression of 5-HT1A_Dro _receptors, but not of any other 5-HT receptor subtype, causes a significant decrease in the response to light of 3^rd ^instar larvae.

**Conclusion:**

Our data demonstrate that activity of serotonergic and corazonergic neurons contribute to the control of larval locomotion by light. We conclude that this control is carried out by 5-HT neurons located in the brain hemispheres, but does not appear to occur at the photoreceptor level and may be mediated by 5-HT1A_Dro _receptors. These findings provide new insights into the function of 5-HT neurons in *Drosophila *larval behavior as well as into the mechanisms underlying regulation of larval response to light.

## Background

Organisms possess a finite number of neuronal networks. Therefore, neurons and circuits must be multifunctional to provide individuals with a variety of behavioral outputs necessary for the adaptation to environmental and developmental changes. Neuromodulation is a powerful way to modify the function of an existing circuit without altering the 'hard-wiring' of such network (reviewed in [1]). In this regard, a large body of evidence indicates that neuromodulatory inputs cause short-term changes on neuronal network activity for adaptation to the environment (reviewed in [2,3]). Moreover, neuromodulators have been shown to play a crucial role in developmental tuning of neuronal circuit function, particularly important for ontogenetic plasticity (reviewed in [4]). In invertebrates, biogenic amines including serotonin and dopamine as well as neuropeptides are well studied neuromodulators regulating a diverse range of physiological, cellular and behavioral processes (reviewed in [5,6]).

*Drosophila *larval locomotion comprises rhythmic waves of forward peristalsis interrupted by episodes of pause, turning and occasional backward crawling [7]. This larval behavior is controlled by the activity of central pattern generators (CPG) [8] and may be modified by neuromodulators such as 5-HT [9] as well as by sensory information including visual input (e.g. [10]; [11]).

*Drosophila *larval visual system consists of two bilateral clusters of 12 photoreceptors each (Bolwig's organs) [12]. Their axons form the larval optic nerve (LON), which projects into the brain toward the larval optic neuropil, also known as larval optic center (LOC) [13]. These photoreceptors express either Rhodopsin 5 (Rh5) or 6 (Rh6) [14], but only Rh5-expressing cells are required for the photophobic response displayed by larvae during foraging stage [15]. This response to light is characterized by increased pausing, head swinging, and direction change as well as reduced linear speed [11,16]. Interestingly, this aversion to light is down-regulated during development, achieving photoneutrality during wandering stage, at which time larvae are searching for a proper site to undergo metamorphosis [17]. The mechanisms underlying the modulation of this behavior remain unknown.

In order to identify neurons that provide neuromodulatory input for the regulation of larval photobehavior, we used genetic tools to study the impact of suppressing synaptic transmission in candidate neurons on the larval response to light. We began by silencing the activity of Ddc-expressing neurons by means of the *Ddc-GAL4 *driver [18]. Ddc catalyzes the last step in the synthesis of both serotonin and dopamine, and thus it is found in both serotonergic and dopaminergic neurons [19]. In addition, it has been reported that a third group of cells, the corazonin (CRZ)-releasing neurons are labeled by the *Ddc-GAL4 *construct during 3^rd ^instar stage [20].

Here, we report that 5-HT neurons located in the brain hemispheres but not those located in the VNC modulate the response to light during larval development. Furthermore, we show that 5-HT signaling is required for proper regulation of larval photobehavior, possibly through activation of 5-HT1A_Dro _receptors. Finally, our findings also suggest that corazonergic neurons may contribute to this modulation.

## Methods

### Fly strains

All *Drosophila melanogaster *stocks were raised at 25°C in standard medium consisting of sucrose, agar 10%, inactivated yeast, and tegosept in ethanol to prevent mold growth. When required, the standard wild type stock Oregon-R (OR) was used. Neuronal silencing experiments were performed using the following *Drosophila *stocks: *w;UAS-TNT (TNT-G*, as the active form, and *TNT-VIF *as the inactive form of tetanus toxin light chain) [21], *yw;UAS-EKO *(or electrically knock out) [22], *w*; *Kr*^*If*-1^*/CyO;UAS-ORK1Δ-C1/TM6C, Sb*^1 ^and *yw;;UAS-ORK1Δ-NC1 *(Bloomington Stock Center, Indiana University, IN, #8928 and #6587 respectively). For cell ablation experiments we used the stock *yw;UAS-hid *[23]. The *w;Ddc-GAL4 *line (HL836, third chromosome) and *w;TH-GAL4 *were a gift from Jay Hirsch (University of Virginia, VA). The line *w;CRZ-GAL4 *[24] was a courtesy of Youn-Jeong Choi (University of Tennessee, Knoxville). The line *w;TRH-GAL4 *used was kindly donated by Barry Condron (University of Virginia Medical School, Charlottesville, VA) [25]. The stock *w;Rh6-GAL4 *was provided by Claude Desplan (New York University, New York, NY). The *w;pBac{PB}CG9122*^*c*01440 ^(or *pBacTRH*) line generated by Exelixis, Inc., was obtained from Wendi Neckameyer (St. Louis University School of Medicine, St Louis, MO). This stock is also available in Bloomington Stock Center, Indiana University, IN (# 10531). The *w;UAS-5-HT1A*_*Dro *_and *w;UAS-5-HT7*_*Dro *_stocks were donated by Julian Dow (University of Glasgow, Glasgow). The *w;UAS-5-HT2*_*Dro *_flies were kindly provided by Luc Maroteaux (Université de Strasbourg, Illkirch). The *w;5HT1B-GAL4/TM3, Ser*, *w;UAS-5HT1B*_*Dro*_*/TM3, Ser *and *yw;UAS-5HT1B*_*Dro*_*RNAi/Cyo *stocks were a courtesy of Amita Sehgal (University of Pennsylvania, Philadelphia). The *eagle *mutant stocks *eg*^*P*289^, *w;eg*^18*B*^*/TM3, Sb*, and *w;eg*^*mz*360 ^(*eg-GAL4*) were kindly donated by Marta Lundell (University of Texas, San Antonio, TX). The line *w;UAS-slit *was provided by Roger Jacobs (McMaster University, ON, Canada). The following stocks were also obtained from Bloomington Stock Center: *w;GMR-GAL4*(# 1104), *w;elav-GAL4 *(# 8765), *w;PBac{GAL4D, EYFP}5-HT2*^*PL*00052 ^(# 19367), and *w;UAS-mCD8:GFP *(# 5137).

### Harvesting and synchronization of larvae

Larvae were harvested following a protocol described previously [26]. Briefly, 4–7 day old parental flies were allowed to mate and lay eggs overnight in fly houses containing food plates (60 mm × 15 mm, Fisher Scientific, Houston, Tx) supplemented with vitamin A (Jamieson Laboratory, β carotene, 1.25 g/L). The next day, following a 2 hour pre-collection, a 1 hour-egg collection was performed. At 21 hours after egg laying (h AEL), all hatched larvae were removed from the collection plate under a dissection microscope. After incubating the remainder of the eggs for a period of 3 hours (corresponding to 21–24 h AEL, or 0–3 h after hatching (AH)), approximately 30–40 newly hatched larvae were collected and transferred to a fresh food plate and allowed to grow until 46–49 h AH ('late 2^nd ^instar'), 65–68 h AH ('early foraging 3^rd ^instar'), 72–75 h AH ('late foraging 3^rd ^instar'), 91–94 h AH ('early wandering 3^rd ^instar stage'), or 96–99 h AH ('late wandering 3^rd ^instar stage').

### Verification of larval stages

Besides performing synchronized larval collections and timing their development at 25°C, several behavioral and anatomical characteristics of the larvae were used to confirm the expected larval stage. Anatomical features that can be used to distinguish the different larval stages include the morphology of the anterior spiracles, the shape of their mouth hooks and the number of teeth [27]. Therefore, these characteristics were checked after every larva was tested. In addition, especially to tell apart foraging from wandering 3^rd ^instar larvae, study of their behaviors such as digging into the food or wandering on the lid of the plate, reversion of spiracles and emptiness of the guts were performed. In this last case, empty guts were verified by disappearance of blue-colored food [28]. For this purpose, food coloring solution (0.05% bromophenol, Sigma) was dissolved in the regular fly medium. Egg collection and larval growth were conducted in the colored-food plate following the same harvesting protocol used for behavioral assays. Animals were removed from their plates at early wandering stage (91–94 h AH) and rinsed with distilled water to remove any excess of food from their bodies. Verification of minimal residual blue staining remaining at the posterior tip of larval guts, characteristic of wandering stage, was performed under a Nikon SMZ1500 light microscope. Lastly, time of pupation was also observed.

### Immunohistochemistry and microscopy

Larval brains were dissected, fixed in 4% paraformaldehyde and incubated with the appropriate primary and secondary antisera as described previously [29]. 5-HT neurons were visualized using rabbit anti-serotonin (1:200) (Protos Biotech Corp., NY). Immunolabeling of larval photoreceptors was performed using the monoclonal 24B10 antibody (1:100), which recognizes the glycoprotein Chaoptin expressed specifically on the photoreceptor-cell plasma membrane [30]. The secondary antibodies used were Texas Red-conjugated goat anti-rabbit IgG (1:200) (Jackson InmunoResearch Laboratories, Inc., West Grove, PA) and Alexa 488-conjugated goat anti-mouse IgG (1:200) (Molecular Probes Inc., Eugene, OR).

Larval brains were viewed in a Nikon Eclipse ∈ 800 microscope. Confocal images were obtained with either a Bio-Rad Radiance MRC 600 Krypton/Argon laser confocal microscope using the LaserSharp software or a Zeiss confocal microscope using LSM510 software. Images were made of z-stack sections and their contrast and brightness were adjusted using Adobe Photoshop 5.0 software for Macintosh.

### Photobehavioral assay and data collection

Photobehavioral assays were conducted using the ON/OFF assay previously used in our laboratory [11]. Larvae were manipulated using a moist paintbrush under a dark room light (20 W lamp with Kodak GBX-2 filter), the same employed for studies of *Drosophila *circadian studies in free running conditions ('constant darkness') [31]. Previous larval photobehavior assays performed in our laboratory using the dark room light as the only light source confirmed earlier observations that *Drosophila *larvae do not respond to light of wavelengths above 650 nm [27].

Prior to the beginning of the photobehavioral assay, single larvae were removed from the food plate, carefully rinsed with distilled water to eliminate any excess of food, and placed on a pre-test non-nutritive agar plate for 1 minute to allow the larva to familiarize with the agar surface. To start the assay, individual larvae were placed then in a test agar plate and subjected to alternative 10 second-pulses of light and dark using a cool white bulb (20W Cool White, Philips) controlled by a tracking program.

Part of the quantitative analysis of larval photobehavior in the ON/OFF assay was conducted using a semi-automatic tracking system previously used in our laboratory [15]. This system allowed for stylus/tablet-based tracking of larval movement. The software (NIH Image 1.62f) automatically calculated a response index, RI = [(total distance traveled (pixels) in the dark period – total distance traveled (pixels) in the light period)/total distance traveled (pixels) in both the periods]. The duration of the assay consisted of a minimum of 60 seconds for foraging larvae and 40 seconds for wandering larvae, with a maximum testing time of 120 seconds in both cases, after which each larva was discarded. All data represented as RIs in figures are depicted as mean ± SEM.

When a detailed quantitative and qualitative analysis of larval behavior in the ON/OFF assay was required, locomotion of new larvae was captured using Pixelink Capture Software and analyzed by means of an advanced tracking software called Dynamic Image Analysis System (DIAS) (3.2; Solltech, Inc., Iowa, USA). This system has been recently used also in our laboratory for a kinematic description of larval locomotion during the ON/OFF assay [11]. Briefly, larval behavior in the ON/OFF assay was recorded for a total time of 60 seconds. The generated digital videos were analyzed in DIAS at a rate of 2 frames per second (2 f/s). Larval outlines were automatically determined using the 'Auto Trace DIC' function and their center positions (centroids) in each frame were automatically calculated. DIAS-based quantitative characterization of larval movement was conducted through analysis of direction change (deg), % of frames in linear locomotion, and centroid translocation (mm), all calculated as described previously [11,32]. Linear locomotion was defined as sequences of at least 5 frames with direction change less than 20 degrees [11,32]. For a qualitative analysis, centroid tracks (series of sequential centroids) and perimeter stacks (changes in larval outlines) of representative larvae were plotted according to their x, y coordinates over the course of the assay.

### Locomotion in constant darkness

The larval response to light as measured in the ON/OFF assay depends on larval locomotion. Therefore, as a control, movement of all larvae used in this study was also examined in constant dark to verify that basic aspects of locomotion were not affected by the genetic background of the larvae. Thus, each larva tested in the ON/OFF assay was also subjected to a 30 second locomotory test using a similar manipulation protocol to the one mentioned above, but in this case under constant safe-light conditions. For quantitative analysis, the assay was performed using the semi-automatic system and data are shown when required as mean number of pixels traveled in 30 seconds () ± SEM. Pixelink Capture Software and DIAS software were used for qualitative description of larval locomotion, and larval centroid tracks and perimeter stacks were generated as described before.

### Touch sensitivity assay

The touch sensitivity test was performed as described in [10] with minor modifications. To avoid bias, this experiment was performed blind. During this assay, general handling of early foraging 3^rd ^instar larvae was the same as during the photobehavioral assay. In this case, larval behavior was observed under a dissection microscope illuminated by a red filter-adapted light source to ensure stimulus-free conditions ('constant darkness'). At the beginning of this assay, single larvae were placed on a non-nutritive agar plate identical to the ones used in the photobehavioral assay and allowed to initiate linear movement. Subsequently, each subject was gently touched with an eyelash on its anterior segments during free-run locomotion. Each larva was touched four times with an interval of 10–15 seconds between strokes. To quantify larval responsiveness to the stimulus, scores 0 to 4 were assigned to the different responses observed. A score of 0 was given to larvae that did not respond to the stimulus, whereas a full stop or hesitation was scored as 1. Larvae that retracted briefly but resumed their forward movement were scored as 2. In those cases in which larvae withdrew their anterior segments followed by a turn away from the stimulus with an angle < 90 degrees, their responses were scored as 3. Finally, when larvae retracted and turned away from the stimulus with an angle > 90 degrees, their behavior was scored as 4. The values obtained for each larva were added, and therefore individual larval scores ranged from 0 to 16. Values are shown as mean () score for each group ± SEM.

### Statistical analysis

Minitab 13.1 software for PC was used for statistical analysis. The statistical tests employed in the analysis of data included one-way analysis of variances (ANOVAs), and Tukey's-pairwise comparisons. Normality test on the residuals of the ANOVAs were conducted using the Rootogram test as well as the Ryan Joiner test. Verification of equal variances of the samples was performed by the F-test or Bartlett's test. In all statistical tests performed, the level of significance α was 0.05.

## Results

### Silencing of Ddc neurons increases the response to light throughout larval development

In order to assess the role of specific neurons in the modulation of larval photobehavior, we used the GAL4/UAS system [33]. In this approach, specific enhancers or promoters are used to regulate the expression of the yeast transcription factor GAL4. A gene of interest, such as tetanus toxin light chain (TNT), is placed under the control of the GAL4-responsive upstream activating sequence (*UAS*), thereby, allowing its expression in a tissue-specific manner [33]. TNT cleaves the vesicle-associated protein synaptobrevin and its targeted expression disrupts evoked neurotransmitter release and decreases spontaneous release by ~50% [21].

The behavioral paradigm used was the ON/OFF assay, in which a single larva placed on a non-nutritive agar surface is subjected to intermittent 10 second-pulses of light. Larval behavior captured during the assay was analyzed by either the software DIAS [11,32,34] or the semi-automatic tracking system [15,26]. Larval behavior during the assay was assessed with DIAS by measuring changes in different locomotory parameters such as centroid translocation, change of direction and amount of linear locomotion that occurs when the larva is exposed to light and dark pulses [11,15]. Segments of linear locomotion have been previously defined as at least 5 consecutive frames with direction change less than 20 degrees per frame (deg/f) [11,32]. The semi-automatic tracking system was used to calculate a response index (RI), using the difference in distance traveled during the dark and light pulses (RI = [(total distance traveled (pixels) in the dark period - total distance traveled (pixels) in the light period)/total distance traveled (pixels) in both periods]).

Previous reports demonstrated that larval locomotion in a non-nutritive substrate is characterized by periods of linear locomotion interspersed by bouts of pause and exploratory behavior (head swinging) followed by a change of path direction [32,34]. Episodes of pause associated with head swinging behavior generate changes in path direction above 20 degrees [11,32,34]. Thus, control of larval locomotion oscillates between two states; one that promotes the peristaltic contraction of the larval musculature leading to linear locomotion and one that triggers episodes of pause and turning. Light dramatically influences the function of this control as seen by the increase in episodes of pause and turning during the light pulse when compared to what is observed during the dark pulse [11]. Interestingly, response to light is markedly reduced by the end of 3^rd ^instar larval stage [17]. The mechanisms responsible for this modulation are currently unknown.

**Figure 1:**
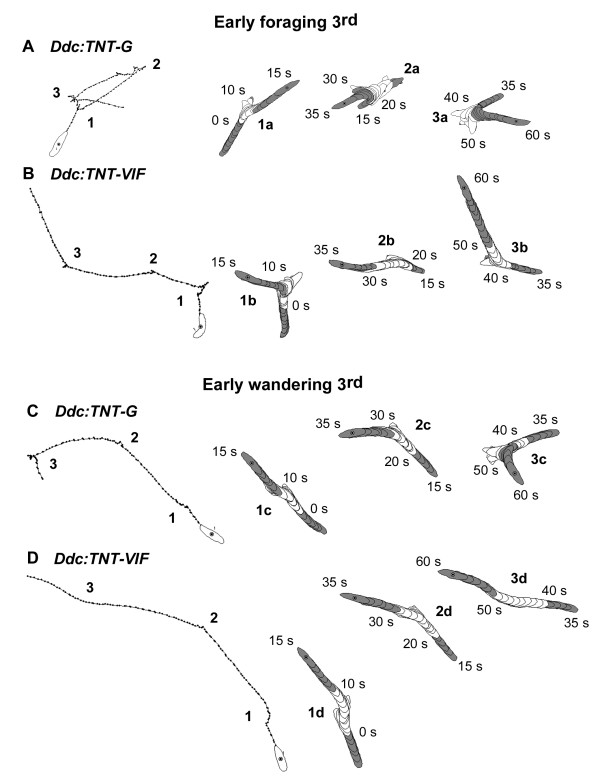
**Representative locomotor patterns during the ON/OFF assay of 3^rd ^instar *Ddc:TNT *larvae**. Centroid tracks (A-D) and perimeter stacks (1–3) were generated using DIAS. A, C, *UAS-TNT-G/+;Ddc-GAL4/+ *(*Ddc:TNT-G*) larvae. B, D, *UAS-TNT-VIF/+;Ddc-GAL4/+ *(*Ddc:TNT-VIF*, control) larvae. In each case, panel 1 represents 5 seconds (s) prior the beginning of the assay and the first 15 s of the assay, panel 2 depicts the following 20 s and panel 3 represents the last 25 s of the assay. Behavior recorded during the light (ON) pulses is shown as empty larval outlines, whereas behavior in the dark (OFF) pulses is shown as shaded larval outlines. Analysis of centroid paths of early 3^rd ^foraging (65–68 h AH) larvae reveals a higher degree of centroid clustering and of irregular centroid arrangement in *Ddc:TNT-G *larvae (A) compared with those in *Ddc:TNT-VIF *larvae (B), particularly during the light (ON) pulses. This suggests that, in the presence of light, *Ddc:TNT-G *larval locomotion is characterized by longer and/or more frequent pausing and change of direction and less linear movement. Inspection of corresponding perimeter stacks further supports these observations. During the light (ON) pulses, foraging *Ddc:TNT-G *larvae exhibited increased head swinging behavior and change of direction when compared with *Ddc:TNT-VIF *larvae (compare 1a-3a with 1b-3b). Although not as pronounced, similar behaviors were observed in *Ddc:TNT-G *larvae during early 3^rd ^instar wandering stage (91–94 h AH) (C), indicating that these larvae still respond to light (1c-3c). In contrast, early wandering *Ddc:TNT-VIF *larvae (D) mostly maintained linear movement and rarely head swung or changed direction over the course of the assay (1d-3d).

**Figure 2:**
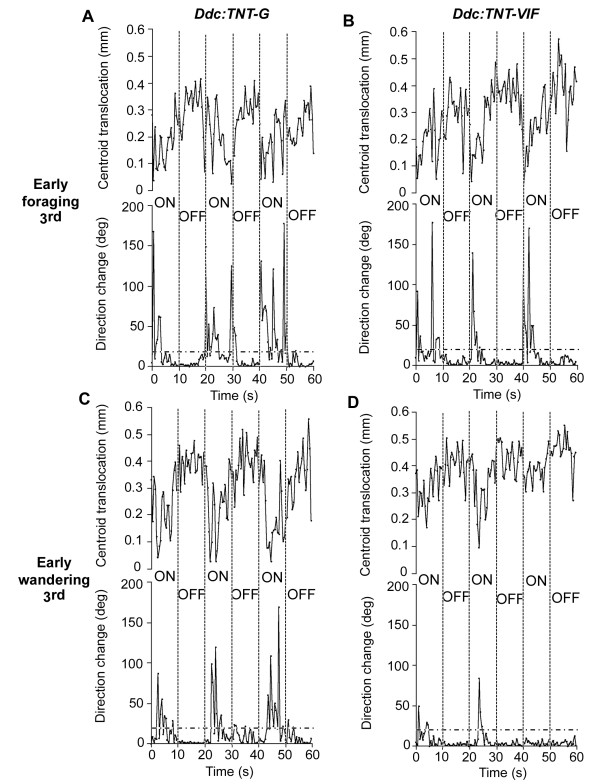
**Centroid translocation and change of direction in 3^rd ^instar *Ddc:TNT *larvae throughout the ON/OFF assay**. Centroid translocation (mm) and direction change (deg) values were obtained from DIAS analyses of the same representative *Ddc:TNT *larvae shown in Fig. 1. A, C, *UAS-TNT-G/+;Ddc-GAL4/+ *(*Ddc:TNT-G*) larvae. B, D, *UAS-TNT-VIF/+;Ddc-GAL4/+ *(*Ddc:TNT-VIF*, control) larvae. Linear movement is defined as segments of at least 5 consecutive frames with changes in direction lower than 20 degrees. Vertical dashed lines delimit the ON/OFF transitions and horizontal dashed lines demarcate the 20 degrees threshold. In the foraging stage (65–68 h AH), light triggers bouts of pausing with little centroid translocation and sharp direction changes (higher than 20 degrees) in both *Ddc:TNT-G *and *Ddc:TNT-VIF *larvae (A and B), although the response appears to be much stronger and more frequent in *Ddc:TNT-G *larvae (A). Furthermore, where a pronounced photophobic response of *Ddc:TNT-G *larvae occurs towards the end of the light (ON) pulse, it appears to persist and finish in the dark (OFF) pulse (e.g. 2^nd ^ON/OFF transition in A). In contrast, during the dark (OFF) pulses both 3^rd ^instar foraging larvae move mostly linearly (i.e. most or all direction change values are lower than 20 degrees), displaying greater centroid translocation. In the wandering stage (91–94 h AH), *Ddc:TNT-G *larvae (C) respond to light. In contrast, during the ON pulses *Ddc:TNT-VIF *wandering larvae (D) maintain linear movement for the most part. Both *Ddc:TNT-G *and *Ddc:TNT-VIF *larvae mostly show linear locomotion during the OFF pulses, similar to what is observed during foraging stage (see also Table 1).

In order to identify potential neuromodulatory inputs that play a role in regulating the larval response to light, we began by silencing the activity of Ddc neurons. To that end, we used the *Ddc-GAL4 *driver [18] to target TNT expression specifically to these cells. Fig. 1 shows centroid tracks and perimeter stacks depicting representative larval behavior during the ON/OFF assay, while Fig. 2 shows the corresponding quantification of locomotion during the assay. During the light pulse, control 3^rd ^instar foraging larvae expressing inactive TNT (TNT-VIF) in Ddc neurons (referred to as *Ddc:TNT-VIF *larvae) present the characteristic head swinging behavior, reduction in centroid translocation and change of direction that lead to reduction of linear movement (Figs. 1B and 2B, [11,32,34]). The behavior of 3^rd ^instar foraging larvae expressing active TNT (TNT-G) in Ddc neurons (henceforth referred to as *Ddc:TNT-G *larvae) during the light pulse is characterized by increased head swinging behavior and change in path direction (Figs. 1A and 2A). Analysis of wandering *Ddc:TNT-VIF *control larvae in contrast shows that, during the light (ON) pulses, these larvae exhibit markedly fewer episodes of head swinging behavior and direction change (Figs. 1D and 2D). Interestingly, wandering *Ddc:TNT-G *larvae respond to light in a manner similar to that of foraging larvae (Figs. 1 and 2, compare C with A and B). The results shown in Fig. 2 are consistent with previous reports that change of direction above 20 degrees are accompanied by sharp reduction in centroid translocation [11,32].

**Figure 3:**
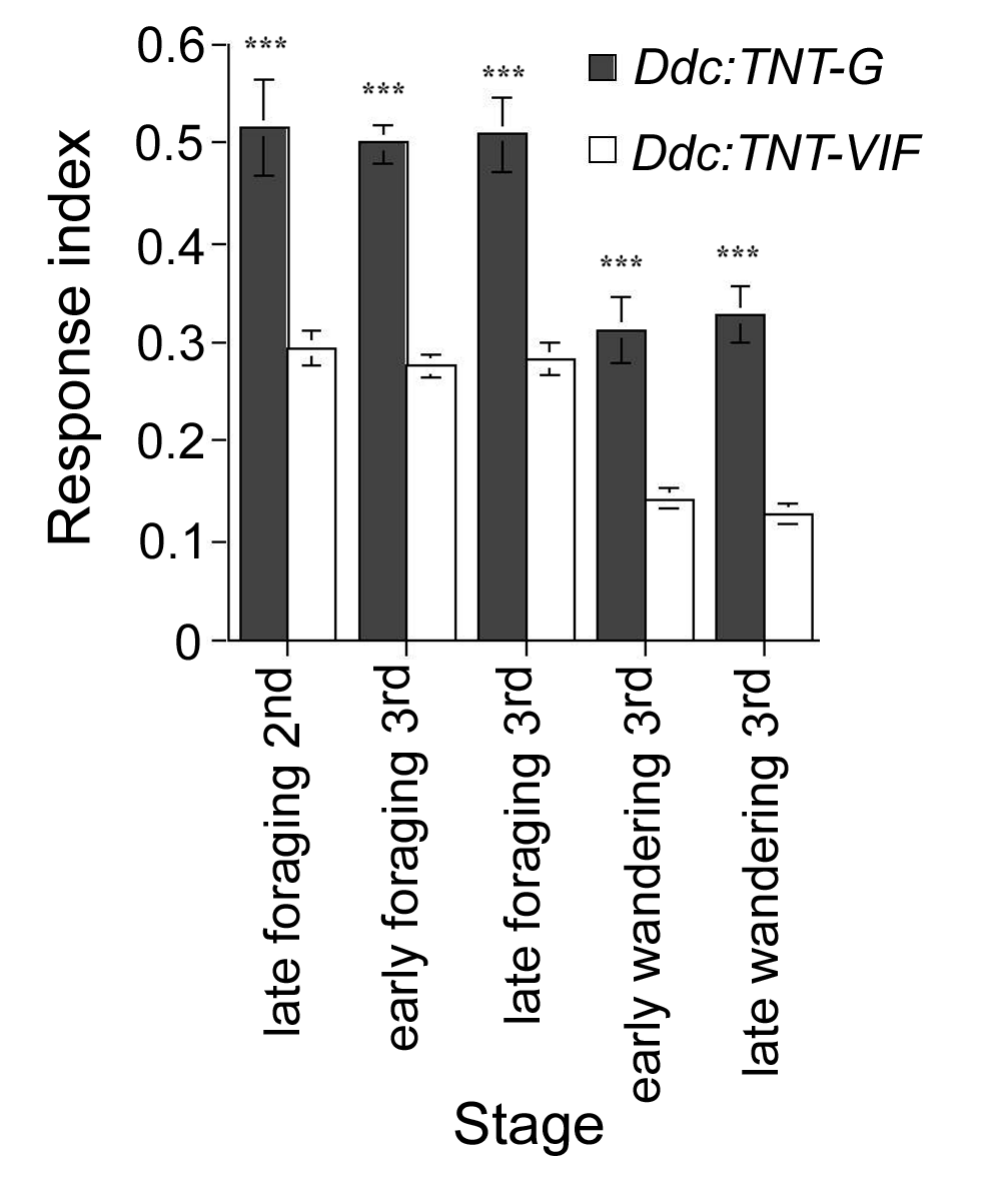
**Larvae expressing active TNT in Ddc neurons present increased response to light throughout development**. Photobehavioral responses measured as Response index (RI) of *Ddc: TNT *larvae tested in the ON/OFF assay at different developmental stages. RI = [(Distance traveled (pixels) in dark (OFF) pulses - distance traveled (pixels) in light (ON) pulses)/Total distance traveled (pixels) during the assay]. In agreement with the results obtain using DIAS, analyses of the RI values obtained using the semi-automatic system show that, compared to control *Ddc:TNT-VIF *larvae, *Ddc:TNT-G *larvae displayed increased response to light throughout larval development (late 2^nd ^instar (46–49 h AH): *UAS-TNT-G/+;Ddc-GAL4/+*, n = 11, RI = 0.52; *UAS-TNT-VIF/+;Ddc-GAL4/+*, n = 12, RI = 0.29; ANOVA: F_(1,21) _= 21.53, p < 0.001; early foraging 3^rd ^instar (65–68 h AH): *UAS-TNT-G/+;Ddc-GAL4/+*, n = 35, RI = 0.50; *UAS-TNT-VIF/+;Ddc-GAL4/+*, n = 23, RI = 0.28; ANOVA: F_(1,56) _= 62.79, p < 0.001; late foraging 3^rd ^instar (72–75 h AH): *UAS-TNT-G/+;Ddc-GAL4/+*, n = 15, RI = 0.51; *UAS-TNT-VIF/+;Ddc-GAL4/+*, n = 14, RI = 0.28; ANOVA: F_(1,27) _= 30.92, p < 0.001; early wandering 3^rd ^instar (91–94 h AH):*UAS-TNT-G/+;Ddc-GAL4/+*, n = 24, RI = 0.31; *UAS-TNT-VIF/+;Ddc-GAL4/+*, n = 23, RI = 0.14; ANOVA: F_(1,45) _= 23.38, p < 0.001; late wandering 3^rd ^instar (96–99 h AH):*UAS-TNT-G/+;Ddc-GAL4/+*, n = 27, RI = 0.33; *UAS-TNT-VIF/+;Ddc-GAL4/+*, n = 24, RI = 0.13; ANOVA: F_(1,49) _= 43.36, p < 0.001). *** p < 0.001.

In order to better understand the changes in light induced-modulation of larval locomotion caused by inactivation of these neurons we used DIAS to measure direction change and amount of linear locomotion during the course of the assay. Table 1 shows that in both genotypes (*Ddc:TNT-G *and *Ddc:TNT-VIF*) linear movement is reduced during the light pulse relative to that measured during the dark pulse. Inactivation of Ddc neurons (*Ddc:TNT-G*) causes an overall reduction of linear locomotion which is much more pronounced during the light pulse. So, while linear locomotion of *Ddc:TNT-VIF *larvae during the light pulses, in comparison to that occurring during the dark pulses, is reduced by 1.3 (foraging) to 1.8 fold (wandering), in *Ddc:TNT-G *larvae this reduction is around 4.1 fold at both stages. Consistent with these results, when the response to light of *Ddc:TNT *larvae was measured in the semi-automated tracking system, a significant increase in the response to light was detected, throughout larval development, in *Ddc:TNT-G *larvae relative to control *Ddc:TNT-VIF *larvae (Fig. 3).

**Table 1:** Parameters of crawling patterns in the ON/OFF assay.

	Early foraging 3^rd^				Early wandering 3^rd^			
	*Ddc:TNT-G* (n = 10)		*Ddc:TNT-VIF* (n = 10)		*Ddc:TNT-G* (n = 8)		*Ddc:TNT-VIF* (n = 6)	
	
	Light	Dark	Light	Dark	Light	Dark	Light	Dark
Frames in linear locomotion (%)	16.61 ± 5.16	68.64 ± 4.94	51.69 ± 4.98	94.41 ± 2.55	18.43 ± 6.52	76.48 ± 5.88	72.88 ± 6.62	97.74 ± 1.49
Direction change (deg/f)	36.49 ± 2.43	12.61 ± 0.95	22.04 ± 2.02	5.19 ± 0.65	32.46 ± 1.65	12.46 ± 2.24	11.06 ± 1.92	4.10 ± 0.47

Sampling rate = 2 f/s. Values are displayed as mean ± SEM.

The values for direction change show a similar trend with a caveat. Upon expression of active TNT in Ddc neurons (*Ddc:TNT-G*), average direction change increases throughout the assay (Table 1). Consistent with previous findings, average direction change is always higher during the light pulses in all genotypes [11]. Interestingly, in foraging *Ddc:TNT-VIF *control larvae average direction change increases 4.2 fold during the light pulses as compared to the dark pulses. In contrast, direction change of *Ddc:TNT-G *foraging larvae is only 2.89 fold higher during the light pulses respect to the dark pulses. Inspection of qualitative and quantitative depiction of the behavior of an individual larva indicates that the increased change of direction during the light pulses caused by expression of active TNT in Ddc neurons may extend beyond the light period into the OFF pulses (e.g. see change of direction during the second ON/OFF transition in Figs. 1A and 2A), which may contribute to the average change of direction calculated for the dark pulse.

Taken together, these observations show that suppression of Ddc neuronal activity causes an increase in light-induced change of direction and associated pause, and thus reduction of linear movement, characteristic of the larval photophobic behavior. Of note is the finding that during wandering stage, inactivation of Ddc-expressing neurons elicits a response to light (Figs. 1C, 2C and 3), whereas wild type larvae and control *Ddc:TNT-VIF *larvae display nearly photoneutral behavior (Figs. 1D, 2D and 3, [17]).

In order to determine whether the silencing of Ddc neurons causes a developmental delay that would explain the elevated larval response to light, all genotypes were tested for developmental timing (see Material and methods). The presence of specific morphological characteristics including the shape of their mouth hooks, the number of teeth, and the morphology of the anterior spiracles as well as behavioral characteristics such as crawling outside the food and emptying of the gut that occurs in the wandering stage demonstrated that suppression of Ddc neuronal activity does not change the timing of larval molts (data not shown) or the onset of the transition from foraging to wandering (see Additional file 1). Finally, we did not observe differences in pupation time between groups (data not shown).

It has been suggested that TNT expression may cause other phenotypes independently from its role as neuronal silencer [35]. Therefore, we carried out similar experiments using genetically modified Shaker and open rectifier K^+ ^channels, EKO and ORK1Δ-C respectively, both previously used to suppress neuronal excitability [36]. Larvae expressing either *UAS-EKO *or *UAS-ORK1Δ-C *construct in Ddc neurons show an increase in their response to light from late 2^nd ^to late 3^rd ^instar stage, similar to that displayed by *Ddc:TNT-G *larvae (see Additional files 2 and 3). Thus, we conclude that neuronal activity of Ddc-expressing neurons is required for regulation of the response to light during larval development.

### Different subsets of Ddc neurons contribute to the modulation of the larval response to light

Ddc catalyzes the last step in the synthesis of both serotonin and dopamine, and thus it is found in both 5-HT and dopaminergic neurons (reviewed in [19]). A third group of cells, the corazonin (CRZ)-releasing neurons are also labeled by the *Ddc-GAL4 *construct [20]. In order to determine which subset (s) of Ddc neuron (s) (serotonergic, dopaminergic or corazonergic neurons) contribute(s) to the increase in the response to light observed in *Ddc:TNT-G *larvae, we took advantage of *GAL4 *driver constructs expressed exclusively in each neuronal type [37].

**Figure 4:**
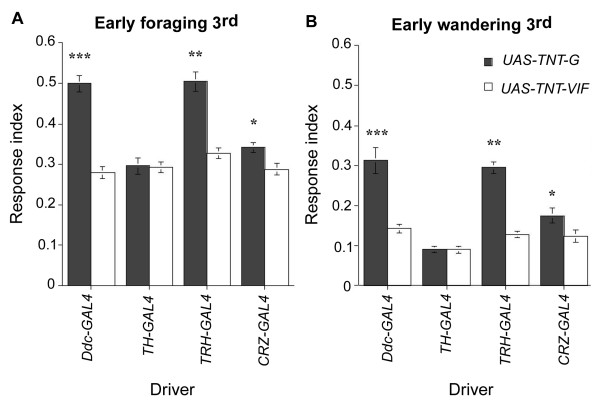
**Response to light of 3^rd ^instar larvae expressing TNT in different subsets of Ddc neurons**. A, Foraging stage. B, Wandering stage. Expression of TNTG under control of *TH-GAL4 *did not affect the larval response to light during 3^rd ^instar, suggesting that dopaminergic neurons do not contribute to the increase in the response to light observed in *Ddc:TNT-G *larvae (early foraging 3^rd ^instar:*UAS-TNT-G/+;TH-GAL4/+*, n = 17, RI = 0.30; *UAS-TNT-VIF/+;TH-GAL4/+*, n = 22, RI = 0.29; ANOVA: F_(1,37) _= 0.02, p = 0.89; early wandering 3^rd ^instar *UAS-TNT-G/+;TH-GAL4/+*, n = 30, RI = 0.09; *UAS-TNT-VIF/+;TH-GAL4/+*, n = 25, RI = 0.09; ANOVA: F_(1,53) _= 0.01, p = 0.91). On the other hand, CRZ neurons appear to partially contribute to the down-regulation of the larval response to light during foraging as well as wandering stage (early foraging 3^rd ^instar:*UAS-TNT-G/+;CRZ-GAL4/+*, n = 20, RI = 0.34; *UAS-TNT-VIF/+;CRZ-GAL4/+*, n = 15, RI = 0.29; ANOVA: F_(1,33) _= 8.34, p < 0.05; early wandering 3^rd ^instar *UAS-TNT-G/+;CRZ-GAL4/+*, n = 22, RI = 0.17; *UAS-TNT-VIF/+;CRZ-GAL4/+*, n = 25, RI = 0.12; ANOVA: F_(1,45) _= 4.59, p < 0.05). Interestingly, *TRH:TNT-G *and *TRH:TNT-VIF *larvae present similar RI values to those observed in *Ddc:TNT-G *and *Ddc:TNT-VIF *larvae (early foraging 3^rd ^instar:*UAS-TNT-G/TRH-GAL4*, n = 21, RI = 0.50; *UAS-TNT-VIF/TRH-GAL4*, n = 21, RI = 0.33; ANOVA: F_(1,40) _= 44.4, p < 0.01; early wandering 3^rd ^instar *UAS-TNT-G/TRH-GAL4*, n = 41, RI = 0.29; *UAS-TNT-VIF/TRH-GAL4*, n = 27, RI = 0.13; ANOVA: F_(1,66) _= 68.31, p < 0.01). *** p < 0.001, ** p < 0.01, * p < 0.05.

To target TNT in dopaminergic neurons, we used the *TH-GAL4 *driver [37]. Tyrosine hydroxylase (TH) performs the rate-limiting step in dopamine biosynthesis and is expressed specifically in dopaminergic cells ([38]; data not shown). Targeted expression of active TNT using the *TH-GAL4 *driver does not cause any change in larval photobehavior (Fig. 4), suggesting that the increase in the response to light seen in *Ddc:TNT-G *larvae is not due to inactivation of dopaminergic neurons.

Tryptophan hydroxylase (TRH, known as TPH in mammals) catalyzes the biosynthesis of 5-hydroxytryptophan from the amino acid tryptophan and constitutes the rate-limiting step in 5-HT production. *Drosophila *has two enzymes able to synthesize 5-HT: neuronal tryptophan hydroxylase (DTRHn, referred here as to TRH), whose expression pattern in the CNS matches that of 5-HT ([39]; data not shown), and phenylalanine hydroxylase (DTPHu), that functions as a non-neuronal or peripheral tryptophan hydroxylase [39-41]. Thus, in order to investigate the possible involvement of 5-HT neurons in modulation of larval photobehavior, we used the *TRH-GAL4 *driver [25].

Expression of TNT-G only in serotonergic neurons (*TRH:TNT-G*) causes a marked increase in the response to light of early foraging and wandering 3^rd ^instar larvae relative to that of control larvae (*TRH:TNT-VIF*) (Fig. 4). Of note, the level of this increase is comparable to that displayed by *Ddc:TNT-G *larvae, suggesting that the increase in the response to light of these larvae may be due mainly to suppression of serotonergic neuronal activity.

In order to evaluate the contribution of CRZ neuronal function to the regulation of larval response to light, we used the *CRZ-GAL4 *driver [24] to target the expression of TNT. *CRZ:TNT-G *larvae show a small but significant increase in the response to light when compared to control *CRZ:TNT-VIF *larvae in both foraging and wandering 3^rd ^instar stages (Fig. 4).

Taken together, these findings indicate that 5-HT neurons but not dopaminergic neurons are involved in the regulation of the larval response to light. In addition, our results suggest that corazonergic neurons also contribute partially to the modulation of this larval behavior during development.

### 5-HT signaling is required for modulation of the larval response to light

**Figure 5:**
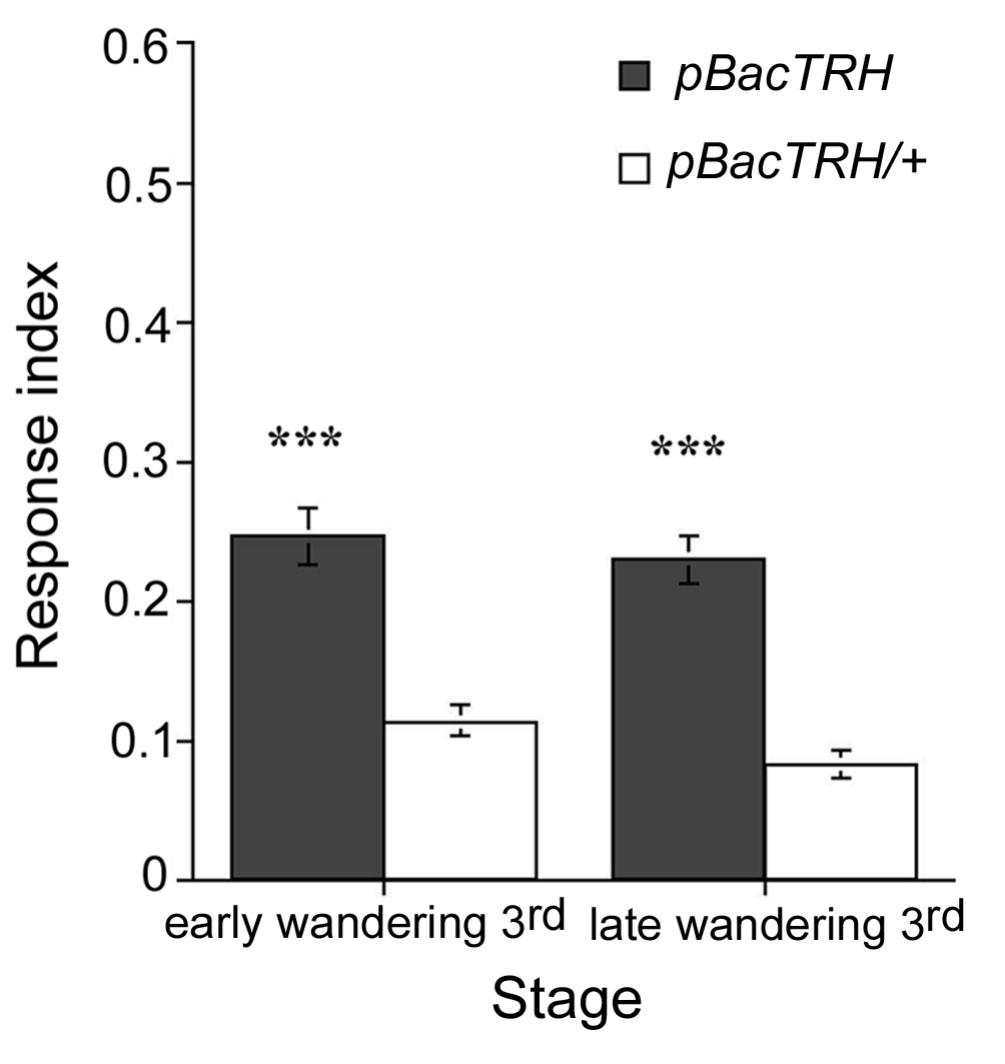
**3^rd ^instar *pBacTRH *mutant larvae display increased response to light**. As measured by their RIs, wandering *pBacTRH *mutant larvae show photophobic response when compared with heterozygous (*pBacTRH/+*) parental control larvae in the same larval stage (early wandering stage: *pBacTRH*, n = 25, RI = 0.25; *pBacTRH/+*, n = 24, RI = 0.11; ANOVA: F_(1,47) _= 35.04, p < 0.001; late wandering stage: *pBacTRH*, N = 25, RI = 0.23; *pBacTRH/+*, n = 24, RI = 0.08; ANOVA: F_(1,47) _= 51.72, p < 0.001). *** p < 0.001.

The results above demonstrate that 5-HT-expressing cells play a role in the modulation of photobehavior during larval development. However, these experiments do not distinguish between this effect being due to decreased release of 5-HT or of other yet unknown neuromodulator also released by these neurons. One way to address this question is by studying the response to light of larvae with reduced 5-HT synthesis. To that end, we analyzed the response to light of homozygous mutant larvae carrying a putative null allele of the *TRH *locus (referred to as *pBacTRH*). These mutant larvae are viable and show diminished 5-HT staining in the CNS [39]. Consistent with our previous observations, *pBacTRH *mutant larvae present an increase in their response to light when compared to that of heterozygous parental control larvae (Fig. 5). Taken together, our observations support the notion that serotonergic neuronal function is required for the modulation of the larval photobehavior. Furthermore, our findings reveal that this modulation is mediated at least in part by 5-HT signaling.

### Silencing of 5-HT neurons does not disrupt larval locomotion

Locomotion represents a task-relevant behavior for the execution of the larval response to light in the ON/OFF assay. Therefore, it is important to determine whether inactivation of serotonergic neurons has an impact on locomotion in general. To that end, we measured the distance traveled by early foraging 3^rd ^instar *TRH:TNT *larvae in constant dark during 30 seconds. *TRH:TNT-G *and *TRH:TNT-VIF *larvae move equally well. Furthermore, no difference was found between the distance traveled by *TRH:TNT-G *and *TRH:TNT-VIF *larvae (*UAS-TNT-G/TRH-GAL4*, n = 27,  = 259.74 ± 4.79 pixels; *UAS-TNT-VIF/TRH-GAL4*, n = 26,  = 266.08 ± 6.87 pixels; ANOVA: F_(1,51) _= 0.58, p = 0.45). In addition, we used DIAS to evaluate the pattern of locomotion of early foraging 3^rd ^instar *Ddc-TNT *larvae in constant darkness. Representative perimeter stacks of *Ddc:TNT-G *larva, similar to that of control *Ddc:TNT-VIF *larva, shows a regular linear arrangement of larval outlines (see Additional file 4). These observations demonstrate that inactivation of serotonergic neurons does not disrupt larval locomotion in our assay.

### Silencing of 5-HT neurons does not increase the response to mechanical stimuli

It is possible that silencing of 5-HT neurons causes increased response to other external stimuli besides light, such as touch. Kernan and colleagues have shown that changes in larval sensitivity to mechanical stimuli can be measured using a touch sensitivity assay [42]. In their assay, wild type 3^rd ^instar larvae present a discrete set of stereotypical responses when stroked with the tip of an eyelash across the anterior body segments during linear locomotion. These responses range from withdrawing from the stimulus and turning away from it to no response at all.

We used a modified version of the touch sensitivity assay [10] to determine whether inactivation of serotonergic neurons also affect the larval response to mechanostimulation. Individual 3^rd ^instar foraging *TRH:TNT *larvae were touched four times during free crawling and the different responses observed were scored using the criteria of Caldwell and collaborators [10] (see also Materials and methods). The scores for each individual larva were added and used to calculate the mean touch response of each larval group (). *TRH:TNT-G *larvae show a small but significant reduction in mechanosensitivity when compared with *TRH:TNT-VIF *larvae (*UAS-TNT-G/TRH-GAL4*, n = 20,  = 5.95 ± 0.37, *UAS-TNT-VIF/TRH-GAL4*, n = 20,  = 7.2 ± 0.35; ANOVA: F_1,38 _= 5.93, p < 0.05). We conclude that synaptic silencing of 5-HT neurons do not cause an overall increase in the response to external stimuli.

### Modulation of the larval response to light requires 5-HT neurons located in the brain hemispheres

A total of 52 serotonergic neurons are found in the VNC in a segmental pattern, forming 14 bilaterally symmetrical clusters: 3 in the subesophageal region, 3 in the thoracic segments and 8 in the abdominal segments [43]. 5-HT projections in each segment, bifurcate ipsilaterally as well as contralaterally, innervating the entire neuropil [20]. Thus, it is possible that modulation of larval photobehavior is carried out by 5-HT neurons located in the VNC.

The zinc-finger transcription factor Eagle (Eg) is required for differentiation of the VNC 5-HT neurons but not for those located in the brain hemispheres [44]. *eg *mutants carrying different alleles display different degree of ablation of VNC 5-HT neurons and the distribution of affected cells appears to be random ([44]; our observations). For instance, larvae homozygous for the *eg*^*P*289 ^hypomorphic allele show severe decrease in the number of 5-HT neurons in both the abdominal and thoracic segments, and to a lesser extent, in the subesophageal region ([44]; see Additional file 5). In contrast, larvae carrying the heteroallelic *eg*^18*B*^*/eg-GAL4 *combination present an overall less drastic reduction in the number of 5-HT-expressing cells of the VNC (see Additional file 5).

**Figure 6:**
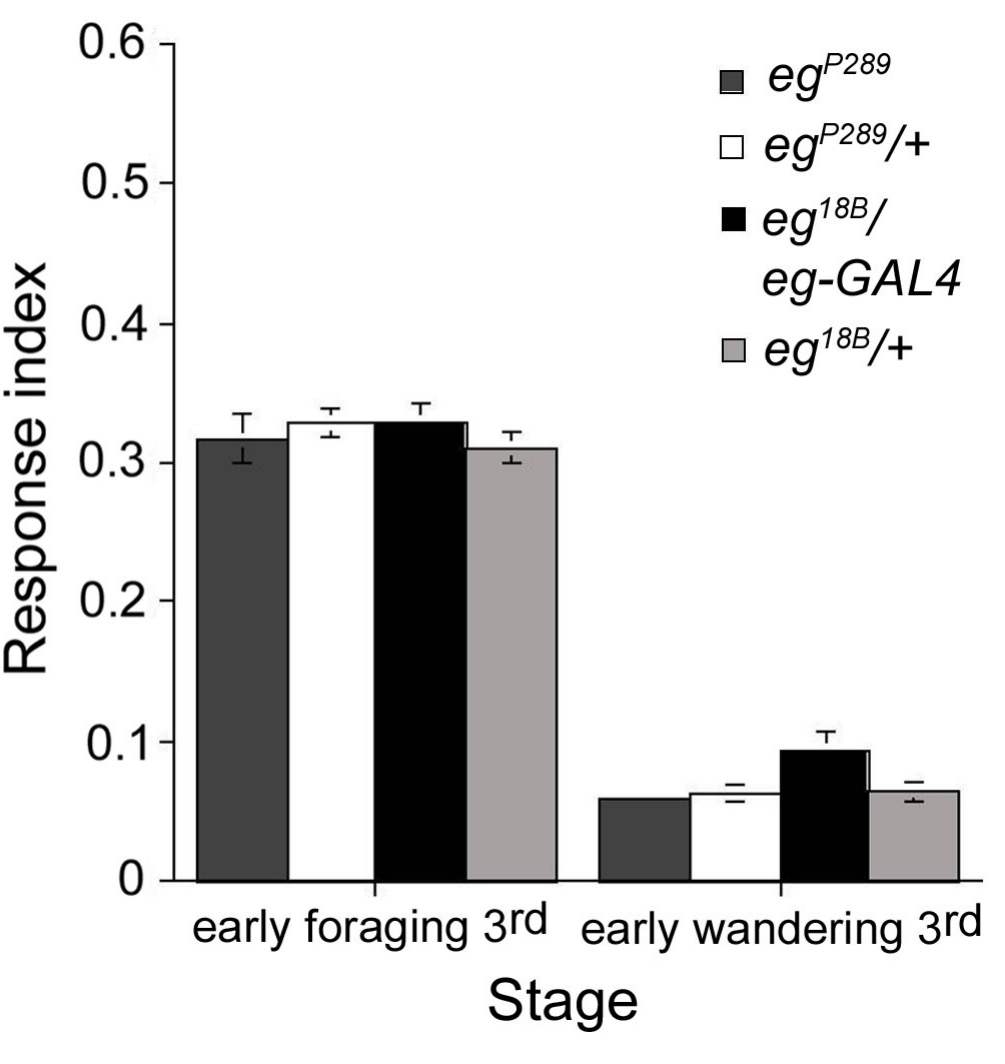
**Lack of VNC 5-HT neurons does not affect the larval response to light**. Photobehavior of foraging and wandering 3^rd ^instar *eg*^*P*289 ^and *eg*^18*B*^*/eg-GAL4 *mutant larvae as well as control larvae during the ON/OFF assay. As measured by their RIs, *eg*^*P*289 ^and *eg*^18*B*^*/eg-GAL4 *larvae show normal photoneutral response when compared with heterozygous parental control larvae in the same larval stage (early foraging 3^rd ^instar:*eg*^*P*289^, n = 21, RI = 0.317; *eg*^*P*289^*/+*, n = 26, RI = 0.328; ANOVA: F_(1,45) _= 0.34, p = 0.561; early wandering 3^rd ^instar *eg*^*P*289^, n = 41, RI = 0.06; *eg*^*P*289^*/+*, n = 25, RI = 0.063; ANOVA: F_(1,64) _= 0.10, p = 0.747; early foraging 3^rd ^instar:*eg*^18*B*^*/eg-GAL4*, n = 19, RI = 0.3283; *eg*^18*B*^*/+*, n = 21, RI = 0.3113; ANOVA: F_(1,38) _= 0.93, p = 0.34; early wandering 3^rd ^instar *eg*^18*B*^*/eg-GAL4*, n = 26, RI = 0.094; *eg*^18*B*^*/+*, n = 21, RI = 0.064; ANOVA: F_(1,45) _= 3.43, p = 0.06).

We used *eg *mutations to evaluate the relative requirement for the larval response to light of serotonergic neurons located in the brain hemispheres versus those located in the VNC. The response to light of *eg*^*P*289 ^and *eg*^18*B*^*/eg-GAL4 *mutants during both foraging and wandering 3^rd ^instar stages is indistinguishable from those of parental control larvae (Fig. 6), demonstrating that 5-HT neurons located in the VNC are not required for regulation of larval photobehavior. Together, these observations point to the 5-HT neurons located in the brain hemispheres as being critical for modulation of the larval response to light.

### 5-HT-mediated modulation of larval photobehavior does not occur at the photoreceptor level

In *Drosophila *larvae, circa 13 5-HT neurons can be seen projecting and arborizing in each brain hemisphere, innervating many different areas of the supraesophageal ganglion including the LOC where it overlaps with the photoreceptor termini [45]. Interestingly, a progressive increase in the innervation of the larval optic neuropil by 5-HT fibers from late 2^nd ^instar to late 3^rd ^instar larval stage coincides with the down-regulation of the larval response to light during this period ([17] and data not shown), suggesting that 5-HT neurons may be exerting their effect at the photoreceptor level.

**Figure 7:**
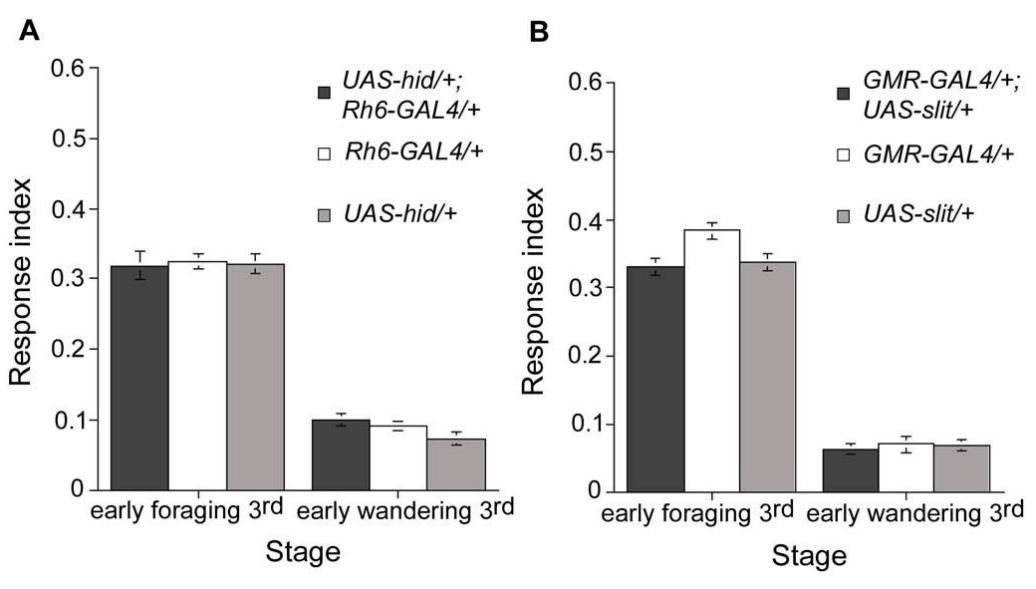
**Disruption of the optic neuropil 5-HT arborization does not affect the larval response to light**. A, Response to light of 3^rd ^instar *UAS-hid/+;Rh6-GAL4/+ *larvae and of *UAS-hid/+ *and *Rh6-GAL4/+ *parental control larvae. B, Response to light of 3^rd ^instar *GMR-GAL4/+;UAS-slit/+ *larvae and parental control *GMR-GAL4/+ *and *UAS-slit/+ *larvae. Consistent with previous results [15], 3^rd ^instar foraging larvae lacking the Rh6 photoreceptors due to targeted expression of *hid *show normal response to light (*UAS-hid/+;Rh6-GAL4/+*, n = 12, RI = 0.32; *Rh6-GAL4/+*, n = 18, RI = 0.32; *UAS-hid/+*, n = 17, RI = 0.32; ANOVA: F_(2,44) _= 0.06, p = 0.95). Similarly, early wandering 3^rd ^instar stage lacking the Rh6 cells displayed the characteristic low response to light (*UAS-hid/+;Rh6-GAL4/+*, n = 22, RI = 0.1; *Rh6-GAL4/+*, n = 24, RI = 0.09; *UAS-hid/+*, n = 21, RI = 0.07; ANOVA: F_(2,64) _= 2.94, p = 0.06). In the case of larvae over-expressing Slit in the larval photoreceptors and their respective control larvae, we found significant differences among the response to light of these strains at early foraging 3^rd ^instar stage (ANOVA: *F*_(2,68) _= 5.77, *p *< 0.05). Nevertheless, *post hoc *analysis of paired mean comparisons revealed that expression of Slit under control of the *GMR-GAL4 *driver caused a small decrease in the larval response to light when compared to the response to light of *GMR-GAL4/+ *larvae but not to that of *UAS-slit/+ *larvae (*GMR-GAL4/+;UAS-slit/+*, n = 20, RI = 0.33; *GMR-GAL4/+*, n = 31, RI = 0.38; *UAS-slit/+*, n = 20, RI = 0.34). At early wandering stage, no differences were found between *GMR-GAL4/+;UAS-slit/+ *larvae and parental controls (*GMR-GAL4/+;UAS-slit/+*, n = 20, RI = 0.06; *GMR-GAL4/+*, n = 13, RI = 0.07; *UAS-slit/+*; n = 20, RI = 0.07; ANOVA: F_(2,50) _= 0.23, p = 0.80).

We have previously reported that ablation of Rh6-specific photoreceptors prevents the appearance of the 5-HT arborization in the larval optic neuropil [29]. Similarly, over-expression of Slit in either all photoreceptors or in the Rh6 subset suppresses branching of the 5-HT processes in the LOC (see Additional file 6). Thus, in order to establish whether innervation of the larval optic neuropil by 5-HT neurons is required for the down-regulation of larval response to light seen during 3^rd ^instar wandering stage, we analyzed the response to light of 3^rd ^instar larvae in which the development of the optic neuropil 5-HT arborization was disrupted either by ablation of the Rh6 photoreceptors (*UAS-hid/+;Rh6-GAL4/+*) or by ectopic expression of Slit (*GMR-GAL4/+;UAS-slit/+*). The results shown in Fig. 7 demonstrate that lack or diminished 5-HT innervation of the LOC does not cause any significant disruption in the larval response to light as measured in the ON/OFF assay. We conclude that 5-HT-mediated regulation of larval photobehavior does not occur at the photoreceptor level.

### 5-HT1A_Dro _is a candidate receptor mediating the serotonergic modulation of the larval response to light

In *Drosophila*, four 5-HT receptors have been identified so far (5-HT1A_Dro_, 5-HT1B_Dro_, 5-HT2_Dro_, and 5-HT7_Dro_). Limited expression data suggest that all receptors are expressed in the CNS throughout *Drosophila *development [46-48]. Mutations are only available for 5-HT1A_Dro _and 5-HT2_Dro _genes [48].

Knowledge of the 5-HT receptor involved in the down-regulation of the larval response to light will aid the identification of neurons critical for the modulation of the larval response to light. Thus, we used a combination of up- and down-regulation approaches in an attempt to identify the candidate receptor/s involved in this phenomenon. For up-regulation, we took advantage of *UAS *constructs available for all receptors identified to date [46,47]. Down-regulation was limitedly achieved by targeted expression of a dsRNA construct available for 5-HT1B_Dro_[46], and a hypomorphic mutation in the 5-HT2_Dro _gene (*5-HT2*^*PL*00052 ^allele) [48]. Although 5-HT1A_Dro _loss-of-function mutant larvae are viable, we were not able to test these larvae as they appear to display a developmental delay phenotype of variable penetrance (data not shown). Pan-neural expression of all *UAS *constructs was achieved by using the *elav-GAL4 *driver.

Based on the results obtained so far, we reasoned that increased 5-HT signaling achieved by up-regulation of 5-HT receptors (5-HT1A_Dro_, 5-HT1B_Dro_, 5-HT2_Dro_, and 5-HT7_Dro_) might reduce the larval response to light during 3^rd ^instar foraging stage. In contrast, if down-regulation of 5-HT signaling by either expression of specific dsRNA constructs (5-HT1B_Dro_) or a single gene mutation (*5-HT2*^*PL*00052^) causes an increase in the response to light this would be likely more noticeable during 3^rd ^instar wandering stage, when normally larvae do not respond to the light stimulus in the ON/OFF assay.

**Figure 8:**
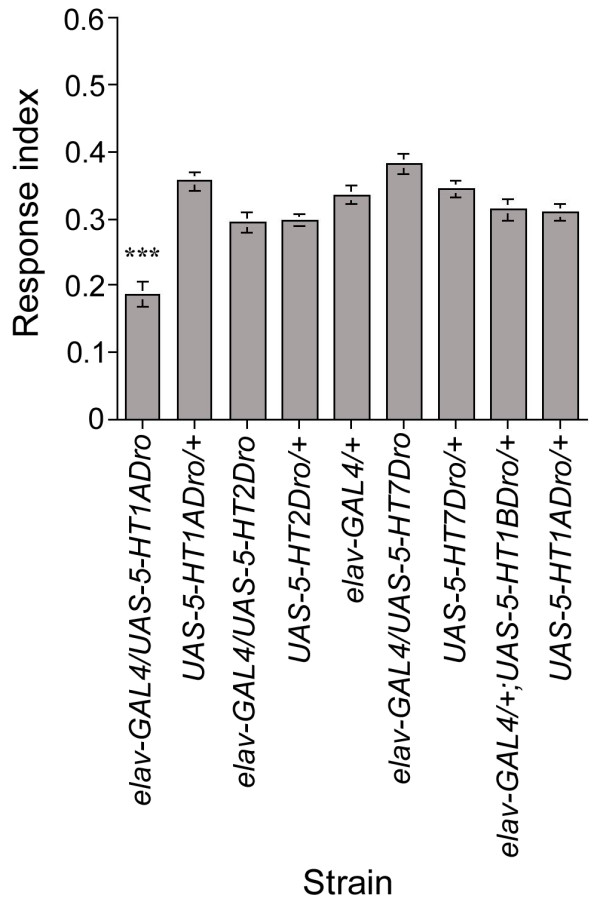
**Pan-neural expression of 5-HT1A_Dro _receptors reduces the larval response to light**. Pan-neural expression of 5-HT1A_Dro_, 5-HT1B_Dro_, 5-HT2_Dro_, or 5-HT7_Dro _receptors in all post-mitotic neurons was carried out by using the *elav-GAL4 *driver. Early foraging 3^rd ^instar larvae expressing 5-HT1A_Dro _receptors but not any of the other 5-HT receptor subtypes in the nervous system showed significantly reduced response to light when compared with both parental control larvae (*elav-GAL4/UAS-5HT1A*_*Dro*_, n = 27, RI = 0.19; *elav-GAL4/+*, n = 20, RI = 0.33; *UAS-5HT1A*_*Dro*_*/+*, n = 30, RI = 0.36; ANOVA: F_(2,74) _= 34.61, p < 0.001). *** p < 0.001.

Using the pan-neural driver *elav-GAL4*, forced expression of 5-HT1A_Dro _receptors, but not of any other 5-HT receptor subtype, causes a significant decrease in the response to light of foraging 3^rd ^instar larvae (Fig. 8). On the other hand, wandering larvae homozygous mutant for the *5-HT2*_*Dro *_gene (*5-HT2*^*PL*00052^) shows the characteristic low response to light when compared with parental controls (data not shown). Similarly, targeted pan-neural expression of the dsRNA construct for the 5-HT1B_Dro _receptor does not affect the response to light of wandering 3^rd ^instar larvae (data not shown). Taken together, these observations point to the 5-HT1A_Dro _receptor subtype as a candidate receptor involved in 5-HT-mediated modulation of the larval response to light.

## Discussion

In *Drosophila *adults, 5-HT neurons are involved in regulation of insulin signaling and organismal growth [49], locomotion [39], aggression [50], circadian rhythms [48], sleep [47], and reproductive function [51]. As well, it has recently been demonstrated that 5-HT neuronal function is necessary for place memory formation [52].

In *Drosophila *larvae, circa 100 5-HT neurons project toward different regions of the CNS, including the LOC, where they contact the LON [29]. Like in other organisms including the adult fly, their broad distribution in the nervous system suggests multiple roles for these cells. For instance, in addition to a suggested role in the modulation of larval heart rate [39], 5-HT neurons have been implicated in regulation of olfactory processing [53], and feeding behavior [39]. Here, we report on the role of serotonergic neurons in the modulation of *Drosophila *larval response to light, a paradigm used to study control of locomotion.

### 5-HT neurons play a role in the modulation of the larval response to light

Larvae in which 5-HT neuronal activity has been suppressed by expression of neuronal silencers (Figs. 1, 2, 3, 4; see Additional files 2 and 3) present an increased response to light during foraging stage as well as partial suppression in the down-regulation of this behavior during the wandering period. The observation that the response to light of *Ddc:TNT-G *larvae is comparable to that of *TRH:TNT-G *larvae should not be simply interpreted as meaning that the only *Ddc-GAL4*-expressing neurons involved in the modulation of larval photobehavior are the serotonergic neurons. Indeed, our findings suggest that CRZ neurons may in part contribute to this regulation (Fig. 4). Differences in the strength of the promoters regulating the various *GAL4 *drivers used for targeted neuronal silencing must be taken into consideration when comparing the contribution of different neuronal groups. Thus, we cannot establish the relative contribution of 5-HT neurons and CRZ neurons to the modulation of the larval response to light. In addition, it has recently been shown that, during 3^rd ^instar larval stage, a fourth group of neurons immunoreactive for crustacean cardioactive peptide (CCAP) and myoinhibiting peptide (MIP) located in the ventral cord are also detected by *Ddc-GAL4 *[43]. Therefore, we cannot exclude the possibility that these cells may also play a modulatory role in the regulation of larval photobehavior.

It is widely accepted that the activity of neuronal networks and the behavioral outputs controlled by them can be regulated by the action of different neuromodulators, which may or may not be co-released by the same terminal. Furthermore, neuronal co-localization of neuropeptides and 'classical' neurotransmitters including biogenic amines has been shown to be quite common in both vertebrates and invertebrates (reviewed in [54]). Therefore, it is important to consider that disruption of neuronal activity of 5-HT cells may affect not only the release of serotonin but also of other transmitter/neuromodulator potentially expressed by these neurons. Although it is currently unknown whether *Drosophila *serotonergic neurons express other neurotransmitters/neuromodulators, previous findings [43] as well as our personal observations (Camilletti and Campos unpublished results) indicate that CRZ and 5-HT do not co-localize.

Behavioral analysis of *TRH *null mutant larvae demonstrates that neuronal 5-HT signaling contributes to modulation of larval photobehavior (Fig. 5). It is important to mention that up-regulation of serotonin synthesis, and presumably therefore its release, by over-expressing TRH in *Ddc-GAL4*-expressing neurons, did not cause a decrease in the response to light of 3^rd ^instar foraging larvae (data not shown and [55]). Nevertheless, this observation does not argue against a role of serotonin in regulation of larval photobehavior. For instance, higher levels of released 5-HT during development might be over-compensated by increased up-take and/or inactivation of this amine as well as by down-regulation of 5-HT receptors, resulting in a wild type behavioral phenotype.

The increased response observed in *pBacTRH *mutants is not as high as that obtained after silencing of 5-HT neurons by TNT expression (compare Fig. 5 with Fig. 4). These results suggest that serotonin may not be the only signaling molecule released by 5-HT neurons and involved in modulation of larval photobehavior. Alternatively, this difference could be explained by residual serotonin release in *pBacTRH *mutants. It has been previously shown that *pBacTRH *larvae show decreased but not complete absence of 5-HT expression in the CNS [39]. These authors suggested that this is due, perhaps, to the re-uptake of circulating 5-HT synthesized peripherally by DTPHu. Thus, it is feasible that, in these mutants, small amounts of 5-HT are still released from serotonergic neurons, thereby partially regulating the larval response to light.

It has been reported that neuronal 5-HT regulates larval feeding [39] and body size in adult flies [49]. Nevertheless, decreased 5-HT levels or release does not appear to affect larval growth, as the size of *Ddc:TNT-G*, *TRH:TNT-G *or *TRH *mutant larvae is within the range of wild type controls (data not shown). These observations are consistent with our conclusion that silencing of the 5-HT neurons did not cause a developmental delay.

As motor performance is crucial for analysis of photobehavior in our assay, it is important to consider the impact of diminished 5-HT synthesis or release on this task-relevant behavior. Previous observations indicate that the locomotion of *TRH *mutant larvae is normal as measured by the number of body wall contractions [39]. Our results agree with those of Neckameyer and collaborators, as *Ddc:TNT-G *(see Additional file 4), *TRH:TNT-G *as well as *TRH *mutant larvae (data not shown) showed normal locomotion in constant dark. In addition, our results demonstrate that inactivation of 5-HT neurons does not result in a generalized disruption of the larval response to external stimuli.

### Modulation of the larval response to light requires 5-HT neurons located in the brain hemispheres

Mutations in the *eg *gene affect serotonergic neurons located in the subesophageal, thoracic and abdominal segments of the VNC but not those 5-HT neurons located in the brain lobes ([56]; see Additional file 5). The remaining VNC 5-HT neurons often show severe pathfinding defects ([44]; see Additional file 5). Interestingly, *eg *mutant larvae respond to light indistinguishably from control larvae and show the expected reduction in this response as they reach the wandering stage, demonstrating that 5-HT neurons located in the VNC are not required in this process (Fig. 6).

It has been shown that CRZ neurons located in the VNC also express *eg *during 3^rd ^instar stage [56]. It is yet to be established whether VNC CRZ neurons are also affected in *eg *mutants. If so, our results suggest that this subset of CRZ cells may not be involved in the modulation of larval photobehavior.

The invasion of the LOC by 5-HT processes and their contact with the LON coincides with the gradual decrease in the larval aversion to light, suggesting that 5-HT neurons may be modulating this larval behavior at the photoreceptor level ([17] and data not shown). However, absence of the 5-HT arborization or disruption of its branching did not affect the response to light of either foraging or wandering larvae (Fig. 7), ruling out 5-HT-mediated modulation of this behavior at the photosensory level.

Neuromodulators may regulate rhythmic motor behaviors by acting at different levels within a specific neuronal circuit, that is, at the sensory and/or central level (reviewed in [3]). The latter may involve modulation within the CPG or at the level of the motorneurons (reviewed in [3]). It is worth noting that the CPG controlling *Drosophila *larval locomotion is thought to be located in the VNC [57]. Thus, one possibility is that the 5-HT neuromodulatory effect occurs within the brain at a central level other than the CPGs (e.g. higher order interneurons). Alternatively, 5-HT neuronal inputs descending from the brain hemispheres may act directly on the CPGs. Interestingly, early immunohistochemical studies have suggested that some 5-HT longitudinal fibers in the VNC may derive from brain lobe neurons [45].

### 5-HT-mediated modulation of larval photobehavior may involve 5-HT1A_Dro _receptors

In both vertebrates and invertebrates, 5-HT is widely expressed and is able to activate several 5-HT receptor subtypes, coupled to different signaling pathways (reviewed in [58]). Our results suggest that 5-HT1A_Dro _receptors may play a role in the modulation of the larval response to light (Fig. 8), further supporting the role of serotonin in this regulation. However, it is important to consider that over-expression of 5HT1A_Dro _receptors using the pan-neural driver *elav-GAL4 *most likely disrupt synaptic activity of the 5-HT neurons themselves. As a result, 5-HT1A_Dro _might act on these cells as an autoreceptor, thus modifying the larval response to light. Therefore, at the present time our observations allow us only to suggest this serotonin receptor as a candidate receptor involved in the regulation of photobehavior. Future experiments aimed at further investigating the possible involvement of this 5-HT_Dro _receptor in this phenomenon will help with the identification of the target cells on which larval 5-HT neurons act to modulate the larval response to light. These cells may in turn represent the critical neurons for the performance of this behavior.

## Conclusion

In this paper we investigated the mechanisms underlying modulation of larval photobehavior and report a novel role for serotonergic and corazonergic neurons in *Drosophila *larva. Our data demonstrate that 5-HT neurons as well as corazonergic neurons contribute to the reduction in the response to light normally observed during larval stage. Study of the serotonergic system indicates that 5-HT-mediated modulation of this behavior is carried out by 5-HT cells located in the brain hemispheres. Furthermore, our observations do not support the idea that this effect is a result of a direct role of 5-HT signaling on photoreceptor termini. Lastly, the suggestion that 5-HT1A_Dro _receptors are involved in this modulation may provide a tool to identify the target neurons of this 5-HT signaling and perhaps critical for the control of locomotion by light.

## Authors' contributions

VGRM carried out the genetic crosses, immunoassays, behavioral studies and performed the statistical analysis. Both VGRM and ARC have participated in the conception and design of the study, as well as with the interpretation of the data. As well, both authors have collaborated in drafting the manuscript. The final version has been approved by both authors.

## Supplementary Material

Additional file 1 **Normal developmental timing of larvae expressing TNT in Ddc neurons**. In order to verify that larvae expressing active TNT in Ddc neurons were wandering at the proper developmental time, emptying of their guts, characteristic of wandering stage, was measured by disappearance of blue-colored food from larval guts. A, B, photographs of representative early wandering *UAS-TNT-G/+;Ddc-GAL4/+ *larva (A) and *UAS-TNT-VIF/+;Ddc-GAL4/+ *larva (B). Early wandering 3^rd ^instar *Ddc:TNT-G *larvae show only residues of blue food at the posterior end of their gut, comparatively similar to what is observed in *Ddc:TNT-VIF *larvae. This suggests that *Ddc:TNT-G *larvae reach the wandering stage at the expected developmental time. Click here for file

Additional file 2 **Expression of ORK1Δ-C in Ddc neurons increases the larval response to light**. Photobehavior in the ON/OFF assay of *Ddc-GAL4/UAS-ORK1Δ-C *(*Ddc:ORK1Δ-C*) and *Ddc-GAL4/UAS-ORK1Δ-NC *(*Ddc:ORK1Δ-NC*, control) larvae tested at different developmental stages. ORK1Δ-C represents a genetically modified constitutively open version of the wild type *Drosophila *open rectifier K^+ ^channel 1 (ORK1). On the contrary, ORK1Δ-NC is a non-conducting version of ORK1Δ-C [36]. RIs were obtained using the semi-automatic tracking system. Compared to what is observed in control larvae, targeted expression of the conductive form of ORK1Δ-C in Ddc neurons increased the larval response to light from late 2^nd ^to late wandering 3^rd ^instar stage (late 2^nd ^instar: *Ddc-GAL4/UAS-ORK1Δ-C*, n = 18, RI = 0.44; *Ddc-GAL4/UAS-ORK1Δ-NC*, n = 15, RI = 0.31; ANOVA: F_(1,31) _= 35.87, p < 0.001; early foraging 3^rd ^instar: *Ddc-GAL4/UAS-ORK1Δ-C*, n = 16, RI = 0.43; *Ddc-GAL4/UAS-ORK1Δ-NC*, n = 15, RI = 0.27; ANOVA: F_(1,29) _= 43.61, p < 0.001; late foraging 3^rd ^instar: *Ddc-GAL4/UAS-ORK1Δ-C*, n = 15, RI = 0.40; *Ddc-GAL4/UAS-ORK1Δ-NC*, n = 17, RI = 0.27; ANOVA: F_(1,30) _= 38.36, p < 0.001; early wandering 3^rd ^instar:*Ddc-GAL4/UAS-ORK1Δ-C*, n = 17, RI = 0.23; *Ddc-GAL4/UAS-ORK1Δ-NC*, n = 17, RI = 0.05; ANOVA: F_(1,32) _= 83.92, p < 0.001; late wandering 3^rd ^instar:*Ddc-GAL4/UAS-ORK1Δ-C*, n = 13, RI = 0.24; *Ddc-GAL4/UAS-ORK1Δ-NC*, n = 17, RI = 0.05; ANOVA: F_(1,28) _= 110.52, p < 0.001). *** p < 0.001. Click here for file

Additional file 3 **Larvae expressing EKO in Ddc neurons show increased response to light**. Photobehavior in the ON/OFF assay of *UAS-EKO/+;Ddc-GAL4/+*, and the parental control *Ddc-GAL4/+ *and *UAS-EKO/+ *larvae tested at different developmental times. The electrically knockout (EKO) represents a genetically modified version of the wild type *Drosophila *Shaker K^+ ^channel [22]. RIs were calculated by the semi-automatic tracking system and statistically analyzed using Tukey's pairwise comparisons. Compared to what is observed in parental control larvae, larvae in which expression of EKO was targeted to Ddc neurons showed increased larval photobehavior from late 2^nd ^to late wandering 3^rd ^instar stage (late 2^nd ^instar: *UAS-EKO/+;Ddc-GAL4/+*, n = 22, RI = 0.44; *Ddc-GAL4/+*, n = 16, RI = 0.37; *UAS-EKO/+*, n = 20, RI = 0.36; p < 0.05; early foraging 3^rd ^instar: *UAS-EKO/+;Ddc-GAL4/+*, n = 17, RI = 0.43; *Ddc-GAL4/+*, n = 12, RI = 0.36; *UAS-EKO/+*, n = 13, RI = 0.33; p < 0.05; late foraging 3^rd ^instar: *UAS-EKO/+;Ddc-GAL4/+*, n = 18, RI = 0.43; *Ddc-GAL4/+*, n = 16, RI = 0.32; *UAS-EKO/+*, n = 18, RI = 0.35, p < 0.05; early wandering 3^rd ^instar:*UAS-EKO/+;Ddc-GAL4/+*, n = 20, RI = 0.19; *Ddc-GAL4/+*, n = 16, RI = 0.06; *UAS-EKO/+*, n = 20, RI = 0.08, p < 0.05; late wandering 3^rd ^instar:*UAS-EKO/+;Ddc-GAL4/+*, n = 20, RI = 0.20; *Ddc-GAL4/+*, n = 15, RI = 0.06; *UAS-EKO/+*, n = 19, RI = 0.11, p < 0.05). * p < 0.05. Click here for file

Additional file 4 **Silencing of Ddc neurons does not affect basic aspects of larval locomotion**. Representative crawling patterns of foraging 3^rd ^instar *Ddc:TNT *larvae in constant darkness. Since the response to light in the ON/OFF assay depends on the ability of larvae to move efficiently, larval locomotion was analyzed during 30 seconds in the absence of light. Perimeter stacks were generated using DIAS. Behavioral analysis using this software shows similar linear movement between *UAS-TNT-G/+;Ddc-GAL4/+ *larvae (A) and control *UAS-TNT-VIF/+;Ddc-GAL4/+ *larvae (B) in constant dark conditions. Click here for file

Additional file 5 ***eagle *mutant larvae present reduced number of 5-HT-expressing neurons in the VNC**. A-F, Confocal micrographs of 3^rd ^instar wandering wild type OR, *eg*^*P*289 ^and *eg*^18*B*^*/eg-GAL4 *mutant brains stained with 5-HT antibody and detected by Texas Red-conjugated secondary. B, D, and F represent the insets of A, C, and E respectively. 5-HT immunolabeling reveals a decreased number of 5-HT neurons in the abdominal (A1–A8) and thoracic segments (T1–T3) as well as in the subesophageal region (SE1–SE3) of CNSs of both *eg*^*P*289 ^and *eg*^18*B*^*/eg-GAL4 *mutants. Note how this phenotype is much more severe in *eg*^*P*289 ^mutants than in *eg*^18*B*^*/eg-GAL4 *mutants. Scale bars in A, C, and E represent 50 μm, whereas in B, D, and F scale bars represent 10 μm. G, Number of 5-HT neurons present in different segments of the VNC of 3^rd ^instar wandering wild type OR larvae, *eg*^*P*289 ^mutant larvae, and heteroallelic *eg*^18*B*^*/eg-GAL4 *mutant larvae. Values are shown as mean ± SEM for each group and as percentages relative to the number found in VNC of OR larvae. Click here for file

Additional file 6 **Expression of Slit in the Rh6 photoreceptors disrupts the proper development of the 5-HT arborization**. A-C, Confocal micrographs of wandering 3^rd ^instar larval brains immunolabeled with anti-5-HT detected by Texas Red-conjugated secondary (red). All larval photoreceptors were immunolabeled with 24B10 monoclonal antibody and detected by Alexa 488-conjugated secondary (A and B, green). Rh6 photoreceptors were labeled by targeted GPF expression (C, green). A, Wild type parental control *UAS-slit/+*. B, *GMR-GAL4/+;UAS-slit/+*. C, *UAS-mCD8-GFP/+;Rh6-GAL4/UAS-slit*. Targeted expression of Slit in either all or only the Rh6 photoreceptors causes a reduction in the development of the 5-HT processes. Scale bars: 10 μm. Click here for file
