# Supplementary figures and images for: Role of serotonergic neurons in the Drosophila larval response to light
Source: BMC Neurosci. 2009 Jun 23;10:66. doi: 10.1186/1471-2202-10-66 (PMC2711092; doi:10.1186/1471-2202-10-66)

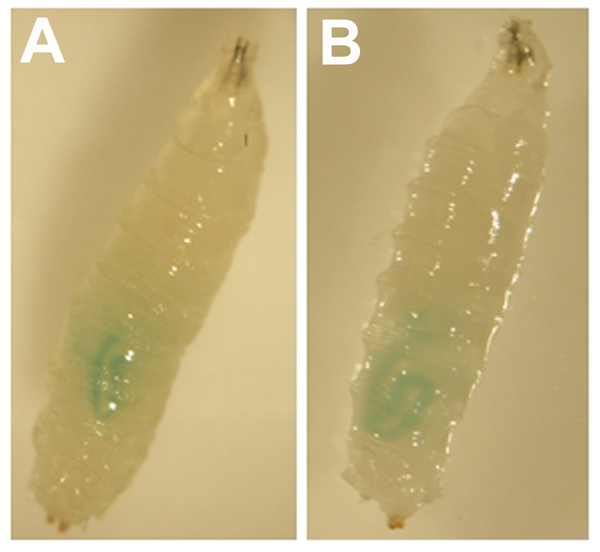

Supplement: Additional file 1 — Normal developmental timing of larvae expressing TNT in Ddc neurons. In order to verify that larvae expressing active TNT in Ddc neurons were wandering at the proper developmental time, emptying of their guts, characteristic of wandering stage, was measured by disappearance of blue-colored food from larval guts. A, B, photographs of representative early wandering UAS-TNT-G/+;Ddc-GAL4/+ larva (A) and UAS-TNT-VIF/+;Ddc-GAL4/+ larva (B). Early wandering 3rd instar Ddc:TNT-G larvae show only residues of blue food at the posterior end of their gut, comparatively similar to what is observed in Ddc:TNT-VIF larvae. This suggests that Ddc:TNT-G larvae reach the wandering stage at the expected developmental time. [file 1471-2202-10-66-S1.tiff]

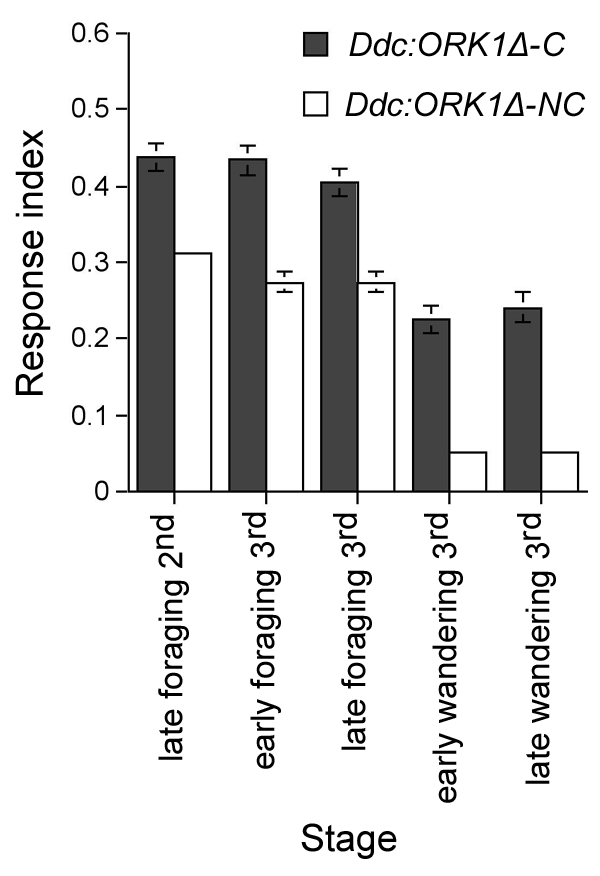

Supplement: Additional file 2 — Expression of ORK1Δ-C in Ddc neurons increases the larval response to light. Photobehavior in the ON/OFF assay of Ddc-GAL4/UAS-ORK1Δ-C (Ddc:ORK1Δ-C) and Ddc-GAL4/UAS-ORK1Δ-NC (Ddc:ORK1Δ-NC, control) larvae tested at different developmental stages. ORK1Δ-C represents a genetically modified constitutively open version of the wild type Drosophila open rectifier K+ channel 1 (ORK1). On the contrary, ORK1Δ-NC is a non-conducting version of ORK1Δ-C [36]. RIs were obtained using the semi-automatic tracking system. Compared to what is observed in control larvae, targeted expression of the conductive form of ORK1Δ-C in Ddc neurons increased the larval response to light from late 2nd to late wandering 3rd instar stage (late 2nd instar: Ddc-GAL4/UAS-ORK1Δ-C, n = 18, RI = 0.44; Ddc-GAL4/UAS-ORK1Δ-NC, n = 15, RI = 0.31; ANOVA: F(1,31) = 35.87, p < 0.001; early foraging 3rd instar: Ddc-GAL4/UAS-ORK1Δ-C, n = 16, RI = 0.43; Ddc-GAL4/UAS-ORK1Δ-NC, n = 15, RI = 0.27; ANOVA: F(1,29) = 43.61, p < 0.001; late foraging 3rd instar: Ddc-GAL4/UAS-ORK1Δ-C, n = 15, RI = 0.40; Ddc-GAL4/UAS-ORK1Δ-NC, n = 17, RI = 0.27; ANOVA: F(1,30) = 38.36, p < 0.001; early wandering 3rd instar:Ddc-GAL4/UAS-ORK1Δ-C, n = 17, RI = 0.23; Ddc-GAL4/UAS-ORK1Δ-NC, n = 17, RI = 0.05; ANOVA: F(1,32) = 83.92, p < 0.001; late wandering 3rd instar:Ddc-GAL4/UAS-ORK1Δ-C, n = 13, RI = 0.24; Ddc-GAL4/UAS-ORK1Δ-NC, n = 17, RI = 0.05; ANOVA: F(1,28) = 110.52, p < 0.001). *** p < 0.001. [file 1471-2202-10-66-S2.tiff]

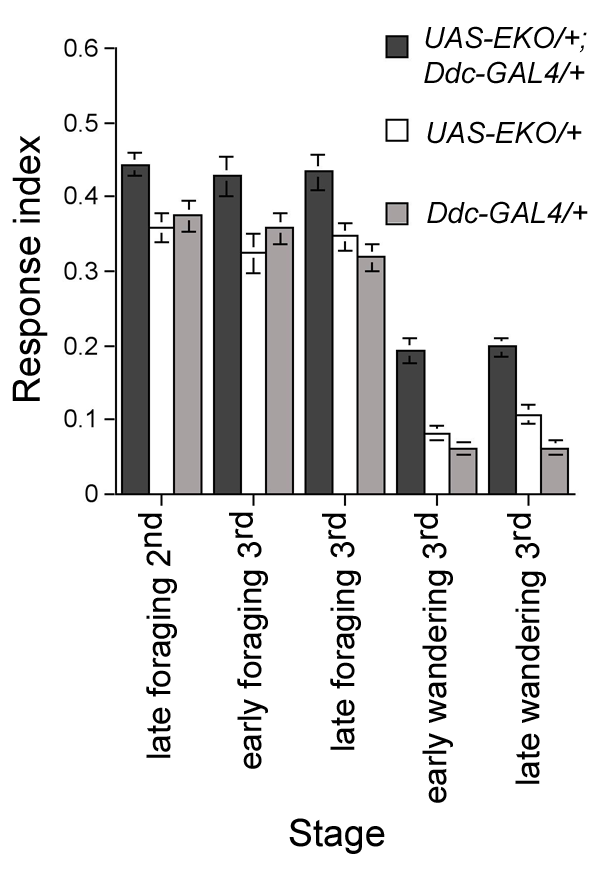

Supplement: Additional file 3 — Larvae expressing EKO in Ddc neurons show increased response to light. Photobehavior in the ON/OFF assay of UAS-EKO/+;Ddc-GAL4/+, and the parental control Ddc-GAL4/+ and UAS-EKO/+ larvae tested at different developmental times. The electrically knockout (EKO) represents a genetically modified version of the wild type Drosophila Shaker K+ channel [22]. RIs were calculated by the semi-automatic tracking system and statistically analyzed using Tukey's pairwise comparisons. Compared to what is observed in parental control larvae, larvae in which expression of EKO was targeted to Ddc neurons showed increased larval photobehavior from late 2nd to late wandering 3rd instar stage (late 2nd instar: UAS-EKO/+;Ddc-GAL4/+, n = 22, RI = 0.44; Ddc-GAL4/+, n = 16, RI = 0.37; UAS-EKO/+, n = 20, RI = 0.36; p < 0.05; early foraging 3rd instar: UAS-EKO/+;Ddc-GAL4/+, n = 17, RI = 0.43; Ddc-GAL4/+, n = 12, RI = 0.36; UAS-EKO/+, n = 13, RI = 0.33; p < 0.05; late foraging 3rd instar: UAS-EKO/+;Ddc-GAL4/+, n = 18, RI = 0.43; Ddc-GAL4/+, n = 16, RI = 0.32; UAS-EKO/+, n = 18, RI = 0.35, p < 0.05; early wandering 3rd instar:UAS-EKO/+;Ddc-GAL4/+, n = 20, RI = 0.19; Ddc-GAL4/+, n = 16, RI = 0.06; UAS-EKO/+, n = 20, RI = 0.08, p < 0.05; late wandering 3rd instar:UAS-EKO/+;Ddc-GAL4/+, n = 20, RI = 0.20; Ddc-GAL4/+, n = 15, RI = 0.06; UAS-EKO/+, n = 19, RI = 0.11, p < 0.05). * p < 0.05. [file 1471-2202-10-66-S3.tiff]

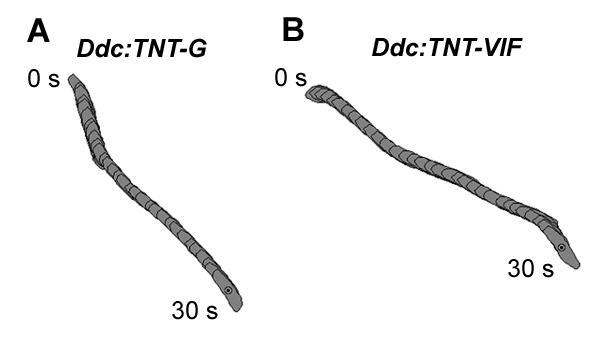

Supplement: Additional file 4 — Silencing of Ddc neurons does not affect basic aspects of larval locomotion. Representative crawling patterns of foraging 3rd instar Ddc:TNT larvae in constant darkness. Since the response to light in the ON/OFF assay depends on the ability of larvae to move efficiently, larval locomotion was analyzed during 30 seconds in the absence of light. Perimeter stacks were generated using DIAS. Behavioral analysis using this software shows similar linear movement between UAS-TNT-G/+;Ddc-GAL4/+ larvae (A) and control UAS-TNT-VIF/+;Ddc-GAL4/+ larvae (B) in constant dark conditions. [file 1471-2202-10-66-S4.tiff]

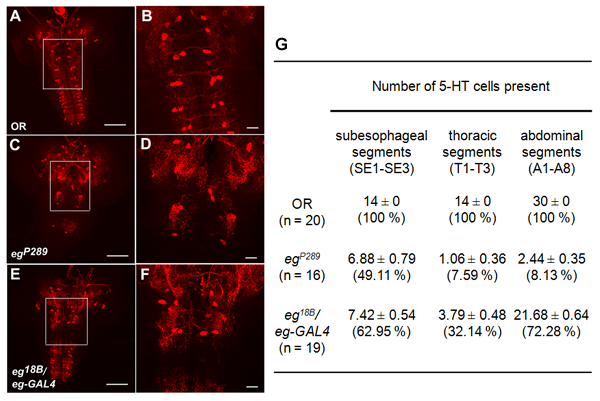

Supplement: Additional file 5 — eagle mutant larvae present reduced number of 5-HT-expressing neurons in the VNC. A-F, Confocal micrographs of 3rd instar wandering wild type OR, egP289 and eg18B/eg-GAL4 mutant brains stained with 5-HT antibody and detected by Texas Red-conjugated secondary. B, D, and F represent the insets of A, C, and E respectively. 5-HT immunolabeling reveals a decreased number of 5-HT neurons in the abdominal (A1–A8) and thoracic segments (T1–T3) as well as in the subesophageal region (SE1–SE3) of CNSs of both egP289 and eg18B/eg-GAL4 mutants. Note how this phenotype is much more severe in egP289 mutants than in eg18B/eg-GAL4 mutants. Scale bars in A, C, and E represent 50 μm, whereas in B, D, and F scale bars represent 10 μm. G, Number of 5-HT neurons present in different segments of the VNC of 3rd instar wandering wild type OR larvae, egP289 mutant larvae, and heteroallelic eg18B/eg-GAL4 mutant larvae. Values are shown as mean ± SEM for each group and as percentages relative to the number found in VNC of OR larvae. [file 1471-2202-10-66-S5.tiff]

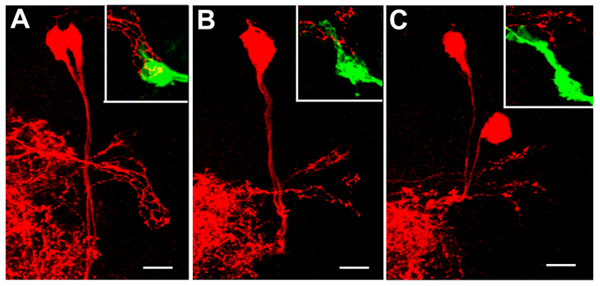

Supplement: Additional file 6 — Expression of Slit in the Rh6 photoreceptors disrupts the proper development of the 5-HT arborization. A-C, Confocal micrographs of wandering 3rd instar larval brains immunolabeled with anti-5-HT detected by Texas Red-conjugated secondary (red). All larval photoreceptors were immunolabeled with 24B10 monoclonal antibody and detected by Alexa 488-conjugated secondary (A and B, green). Rh6 photoreceptors were labeled by targeted GPF expression (C, green). A, Wild type parental control UAS-slit/+. B, GMR-GAL4/+;UAS-slit/+. C, UAS-mCD8-GFP/+;Rh6-GAL4/UAS-slit. Targeted expression of Slit in either all or only the Rh6 photoreceptors causes a reduction in the development of the 5-HT processes. Scale bars: 10 μm. [file 1471-2202-10-66-S6.tiff]
